# Nutritional genomics, inflammation and obesity

**DOI:** 10.20945/2359-3997000000255

**Published:** 2020-06-05

**Authors:** Telma Angelina Faraldo Corrêa, Bruna Jardim Quintanilha, Marina Maintinguer Norde, Marcela Augusta de Souza Pinhel, Carla Barbosa Nonino, Marcelo Macedo Rogero

**Affiliations:** 1 Departamento de Alimentos e Nutrição Experimental Faculdade de Ciências Farmacêuticas Universidade de São Paulo São Paulo SP Brasil Departamento de Alimentos e Nutrição Experimental , Faculdade de Ciências Farmacêuticas , Universidade de São Paulo (USP), São Paulo , SP , Brasil; 2 Centro de Pesquisa em Alimentos Centros de Pesquisa, Inovação e Difusão Fundação de Amparo à Pesquisa do Estado de São Paulo São Paulo SP Brasil Centro de Pesquisa em Alimentos (FoRC), Centros de Pesquisa, Inovação e Difusão (Cepid), Fundação de Amparo à Pesquisa do Estado de São Paulo (Fapesp), São Paulo , SP , Brasil; 3 Departamento de Nutrição Faculdade de Saúde Pública Universidade de São Paulo São Paulo SP Brasil Laboratório de Genômica Nutricional e Inflamação, Departamento de Nutrição , Faculdade de Saúde Pública , Universidade de São Paulo (USP), São Paulo , SP , Brasil; 4 Departamento de Medicina Interna Faculdade de Medicina de Ribeirão Preto Universidade de São Paulo Ribeirão Preto SP Brasil Departamento de Medicina Interna , Faculdade de Medicina de Ribeirão Preto , Universidade de São Paulo (USP), Ribeirão Preto , SP , Brasil; 5 Departamento de Ciências da Saúde Faculdade de Medicina de Ribeirão Preto Universidade de São Paulo Ribeirão Preto SP Brasil Departamento de Ciências da Saúde , Faculdade de Medicina de Ribeirão Preto , Universidade de São Paulo (USP), Ribeirão Preto , SP , Brasil

**Keywords:** Genetic polymorphism, Mediterranean diet, monounsaturated fatty acids, omega-3 fatty acids

## Abstract

The Human Genome Project has significantly broadened our understanding of the molecular aspects regulating the homeostasis and the pathophysiology of different clinical conditions. Consequently, the field of nutrition has been strongly influenced by such improvements in knowledge – especially for determining how nutrients act at the molecular level in different conditions, such as obesity, type 2 diabetes, cardiovascular disease, and cancer. In this manner, characterizing how the genome influences the diet and vice-versa provides insights about the molecular mechanisms involved in chronic inflammation-related diseases. Therefore, the present review aims to discuss the potential application of Nutritional Genomics to modulate obesity-related inflammatory responses. Arch Endocrinol Metab. 2020;64(3):205-22

## NUTRITIONAL GENOMICS

The Human Genome Project (HGP), formally launched in 1990 and finished in 2003, triggered a relevant foundation for research in the health field. However, translating whole genome sequencing into therapies that will benefit an individual will require strategies to handle large amounts of biological and medical data and the ability to identify significant and clinically meaningful results. It should be noted that nutrition science was strongly influenced by HGP, through the consolidation of Nutritional Genomics ( 1 - 3 ).

Nutritional Genomics is a field of nutrition science that encompasses areas such as Nutrigenomics, Nutrigenetics, and Nutritional Epigenomics. These subjects address the interactions between the environment, nutrients, bioactive compounds in foods, and genes, as well as how these interactions influence phenotype, including disease-development risks ( 1 ).

Nutrigenomics studies aim at verifying how gene expression is regulated by nutrients and food components, since specific nutrients and food components may increase or decrease the expression of a given gene. In this manner, interactions between such nutrients and genes may occur either through direct or indirect means. Regulation through indirect means occurs by the ability of nutrients and bioactive compounds to activate intracellular signaling pathways. Intracellular signaling activation, in turn, promotes the translocation of specific transcription factors from the cytoplasm to the cell nucleus. Several transcription factors bind to the promoter region of specific genes to induce gene transcription ( 2 ). On the other hand, regulation through direct means involves the direct interaction between nuclear receptors (or transcription factors) and nutrients or bioactive compounds, whose fact promotes changes in gene expression ( 3 - 5 ).

Genetic variability, *i.e.* , the differences in the sequence of nucleotides, influences how individuals interact with environmental factors. Therefore, Nutrigenetics assesses the influence of individual genetic variability to that of diet and the resultant risk of developing nutrition-related diseases. Nutrigenetics encompasses studies on variations observed in DNA, such as single nucleotide polymorphisms (SNP), copy number variation (CNV), and insertions and deletions (INDELs) ( 1 , 2 , 6 ). In addition, Nutritional Epigenomics is the third subarea within Nutritional Genomics. This area deals with assessing the influence of diet on epigenetic mechanisms that regulate gene activity and expression. Epigenetics encompasses DNA methylation, histone modifications (histone methylation, acetylation, and phosphorylation), and noncoding RNA activity (mainly microRNAs).

## NUTRIGENOMICS, INFLAMMATION, AND OBESITY

Obesity is related to chronic low-grade inflammation. Macrophages and T cells infiltrate the adipose tissue stimulating the release of inflammatory molecules, such as tumor necrosis factor-α (TNF-α), plasminogen activator inhibitor-1 (PAI-1), interleukins (IL-6, IL-1β, IL-8) and inflammatory modulators, such as leptin, resistin, and adiponectin ( 7 ). Chronic inflammation disrupts a whole range of metabolic pathways – these being insulin signaling, glucose homeostasis, and lipid metabolism.

Dietary intake and nutritional status are relevant environmental factors that can modulate metabolic inflammation. Western diets rich in saturated fatty acids (SFA), sugar, and refined grains are linked to cardiovascular diseases, type 2 diabetes (T2D), obesity, and other metabolic disorders. Moreover, Western diets may increase postprandial expression of proinflammatory cytokines and the nuclear factor kappa B (NF-kB) activation in human peripheral blood mononuclear cells (PBMC) ( 8 , 9 ). NF-kB can regulate the expression of adhesion molecules in response to inflammatory stimuli such as E- and P-selectins, intercellular adhesion molecule-1 (ICAM-1), and vascular cell adhesion molecule-1 (VCAM-1). NF-kB also induces the expression of proinflammatory cytokines, including IL-6 and TNF-α ( 10 ).

Conversely, the Mediterranean diet (MedDiet) is rich in monounsaturated fatty acids (MUFA) and polyphenols ( 9 ). In a study conducted by Esmaillzadeh and cols. ( 11 ), a dietary pattern similar to MedDiet reduced plasma C-reactive protein (CRP) and sVCAM-1 levels, while the Western diet increased plasma serum amyloid A (SAA) and IL-6 levels. The results indicated that dietary patterns are associated with the plasma concentration of inflammatory biomarkers. The main inflammatory biomarkers related to obesity are shown in Table 1 .

**Table 1:** Main inflammatory biomarkers in obesity-associated low-grade inflammation and associated mechanisms

Population	Inflammatory biomarkers	Mechanisms	Ref.
Obese subjects	TNF-α	M1 macrophages infiltration in the adipose tissue → ↑ TNF-α which binds to its receptor (TNFR1) → NF-kB and AP-1 activation → ↑ pro-inflammatory cytokines production.	( 24 )
Obese subjects submitted to bariatric surgery	IL-6 and hsCRP	TNF-α and IL-1 → ↑ IL-6 which is produced from several sites, including adipose tissue. IL-6 induces hepatic synthesis of CRP and fibrinogen. Weight loss: ↓ IL-6 and CRP.	( 130 )
Obese women who underwent a 4-week caloric restriction (800 kcal/day)	hsCRP	Weight loss reduced gut permeability and LBP level, possibly reducing plasma hsCRP.	( 131 )
Healthy subjects and subjects with T2D submitted to an oral glucose tolerance test (OGTT)	hsCRP, IL-6, TNF-α, sICAM-1, sVCAM-1, and sE-selectin	Glucose load: ↑ biomarkers of low-grade inflammation in both groups. Subjects with T2D: higher increase in TNF-α and sE-selectin.	( 132 )
Healthy subjects who received an oral fat load	sICAM-1, sVCAM-1, hsCRP, sE-selectin, IL-6, TNF-α	Fat load: ↑ inflammatory biomarkers. Free fatty acids activate pro-inflammatory serine kinase cascades (IkB kinase and c-Jun N-terminal kinase) which stimulate adipose tissue to release IL-6.	( 133 )
Elderly subjects at high risk for cardiovascular disease	IL-6, IL-8, MCP-1	Long-term adherence to MedDiet reduced plasma inflammatory biomarkers probably due the inhibition of the NF-kB pathway activation by MedDiet polyphenols.	( 13 )

AP-1: hsCRP: high sensitivity C-reactive protein; IL: interleukins; LBP: lipopolysaccharide binding protein; MCP-1: monocyte chemoattractant protein-1; NF-kB: nuclear factor kappa B; sE-selectin: soluble E-selectin; sICAM: soluble intercellular adhesion molecule-1; sVCAM-1: soluble vascular cell adhesion molecule-1; T2D: type 2 diabetes; TNF-α: tumor necrosis factor alpha. ↑: increase.

### Mediterranean diet and gene expression

MedDiet is characterized by high consumption of olive oil, fruits, vegetables, whole grains, beans, nuts, seeds, and legumes. It also involves a moderate consumption of fish and wine, as well as a low intake of red meat, sweets, and dairy products ( 12 ). Such diet exerts anti-inflammatory and immunomodulating activities, thus decreasing pro-inflammatory molecules such as interleukins (IL-6, IL-8, IL-18), TNF-α and its receptor, CRP, monocyte chemoattractant protein-1 (MCP-1), and endothelial adhesion molecules (VCAM-1, ICAM-1, and E- and P-selectins) ( 13 ). Accordingly, studies have also shown that MedDiet may reduce the risk of disorders related to oxidative stress, chronic inflammation, and the immune system ( 9 , 13 ).

In a clinical trial conducted by Camargo and cols. ( 9 ), the effects of dietary fat on the postprandial proinflammatory gene expression were verified in twenty elderly subjects. The authors observed that a MedDiet enriched in MUFA with virgin olive oil downregulated *NF-kB p65* gene expression and up-regulated *IkB* α gene expression in PBMC when compared with SFA-rich and low-fat diets. The low-fat regimen was a high carbohydrate, n-3 polyunsaturated fatty acid-enriched diet (CHO-PUFA). Regarding proinflammatory cytokines, the individuals on MedDiet presented a downregulation of *MCP-1* and *TNF-* α gene expression when compared with SFA and CHO-PUFA diets, respectively.

The PREDIMED study assigned elderly subjects (55-80 years of age) at high cardiovascular risk to three diets: either the MedDiet supplemented with extra virgin olive oil (EVOO), MedDiet supplemented with nuts, or a low-fat control diet ( 14 ). In the PREDIMED population, MedDiet supplemented with EVOO attenuated the increase in cyclooxygenase-1 ( *COX-1* ) and low-density lipoprotein receptor-related protein ( *LRP1* ) gene expression. In human monocyte-derived macrophages, MedDiet resulted in a decreased *MCP-1* gene expression when compared to either MedDiet supplemented with nuts, or a control diet ( 15 ).

The effects of both the MedDiet and the Western diet were tested by replacing SFA with MUFA in abdominally obese men and women for eight weeks. Consumption of MUFA on MedDiet attenuated oxidative phosphorylation gene expression, plasma connective tissue growth factor, and apolipoprotein B levels in PBMC when compared to the SFA diet. The MUFA diet also modulated gene expression involved in B-cell receptor signaling and endocytosis. The MedDiet group showed reduced plasma levels of pro-inflammatory proteins, such as IL-1β, macrophage inflammatory protein 1-α, serum amyloid P, and vascular endothelial growth factor (VEGF) ( 16 ).

### Olive oil and inflammation in obesity

In the MedDiet, olive oil (especially EVOO) is the primary source of dietary lipids and is rich in MUFA – especially oleic acid and phenolic compounds. Olive oil is also a bioactive food, which may be responsible for anti-atherogenic, anti-inflammatory, anti-diabetes, and immunomodulatory activity ( 12 , 17 ).

Olive oil consumption is related to improvements in lipid profile, insulin resistance, oxidative damage, inflammatory biomarkers, endothelial function, and blood pressure. Some of these effects are dose-dependent on olive oil’s phenolic content ( 18 - 20 ). Furthermore, olive oil has also been linked to decreased levels of inflammatory biomarkers such as IL-6, CRP, E- and P-selectin, sVCAM-1, and sICAM-1 ( 12 ). Bioactive compounds of olive oil may modulate different levels of gene expression, such as transcription, maturation, and stability of RNA – in addition to translation in proteins, and other post-transcriptional events ( 19 ).

The composition and concentration of polyphenols in olive oil, as well as their bioavailability and how it is metabolized in the human body, are all essential to determine their health effects. The intake of olive oil in the MedDiet is 30 to 50 g/day, which results in an intake of 4-9 mg/day of polyphenols ( 21 ). Olive oil has over 30 polyphenols, of which oleuropein and hydroxytyrosol may influence obesity-related genes. Hydroxytyrosol can modulate genes related to adipocyte maturation and differentiation. This polyphenol is also responsible for inhibiting lipid synthesis. In addition, hydroxytyrosol and oleuropein may reduce the fat-cell size and, consequently, decrease the risk of obesity. In this context, polyphenols in adipose tissue can downregulate genes related to adipogenesis such as peroxisome proliferator-activated receptor ( *PPAR)* γ, *CCAAT enhancer-binding protein-* α ( *C/EBP* α), and *sterol regulatory element-binding transcription factor 1c* ( *SREBP-1c* ) transcription factors. This is also the case for downstream genes such as *CD36, FASN,* and *glucose transporter 4* ( *GLUT4* ) ( 21 ).

EVOO polyphenols can also reduce the risk of metabolic syndrome. In a study conducted with abdominally overweight subjects at risk of metabolic syndrome, the replacement of SFA by MUFA (olive oil) led to a greater anti-inflammatory gene expression profile in adipose tissue. Considering that adipose tissue has a crucial role in lipid metabolism and inflammation, the replacement of SFA by MUFA prevented adipose tissue inflammation and consequently reduced the risk of inflammatory diseases, such as metabolic syndrome ( 22 ).

### Eicosapentaenoic acid (EPA) and docosahexaenoic acid (DHA)

EPA and DHA may attenuate obesity-related inflammation. The proposed mechanism for this effect is the inhibition of the NF-kB in several tissues by activating PPAR-γ and other signaling proteins ( 23 ). PPAR-γ activation decreases the expression of genes that code for pro-inflammatory proteins through the inhibition of NF-kB activation. Also, EPA and DHA present another mechanism to modulate the inflammatory response by binding to G-protein coupled receptor 120 (GPR120). GPR120 activation induced by EPA or DHA leads to the reduced expression of genes with pro-inflammatory actions, such as *TNF-* α and *IL-6.* The interaction between EPA, DHA, and PPARs modulates the expression of genes involved in lipid metabolism, thus reducing both adipogenesis and fat deposition in the adipose tissue ( 24 ).

A recent clinical trial compared the effects of EPA and DHA on inflammatory biomarkers in subjects with abdominal obesity and subclinical inflammation. Participants were supplemented with capsules containing either EPA (2.7 g/day), DHA (2.7 g/day), or corn oil (3 g/day; control) for eight weeks. Both EPA and DHA were more effective than corn oil in reducing inflammation. However, DHA was more potent in modulating inflammation biomarkers in comparison to EPA. In this way, DHA induced a more significant reduction of serum IL-18 and greater increased adiponectin compared with EPA. DHA also reduced plasma levels of CRP, IL-6, IL-18, and TNF-α while increased plasma adiponectin levels when compared to the control ( 23 ).

In another study from the same group ( 25 ), abdominal obesity and low-grade inflammation subjects were supplemented with the same amounts of EPA, DHA, or corn oil for ten weeks. The authors did not observe any difference between EPA and DHA in the expression of inflammation-related genes in whole blood cells. However, EPA increased *PPAR* α expression and reduced *CD14* expression relative to the control, while DHA upregulated the expression of *PPAR* α and *TNF* α, and downregulated *CD14* expression. The supplementation with DHA (3 g/day) for ten weeks also decreased gene expression and secretion of TNF-α and MCP-1, while EPA increased serum IL-10 and reduced *TNF* α expression in monocytes of subjects with chronic inflammation ( 26 ). Despite the beneficial effects on inflammatory biomarkers, DHA had increased plasma LDL-c ( 26 ). Evidence also indicates that DHA is more potent than EPA in increasing LDL-c concentrations ( 27 ).

The effect of olive oil on gene expression was also compared to EPA and DHA. Subjects with mildly elevated plasma lipoprotein-phospholipase A2 were supplemented with either olive oil (6 g/day), EPA (1.8 g/day), or DHA (1.8 g/day) for six weeks. Only EPA supplementation was associated with changes in gene expression in the IFN pathway and downregulation of *cyclic adenosine monophosphate* ( *cAMP* ) *responsive element protein 1* ( *CREB1* ) and *hypoxia-inducible factor 1 alpha subunit* ( *HIF1A* ) ( 28 ).

## NUTRIGENETICS AND OBESITY

An individual’s genetic profile may influence the sensitivity to the development of obesity ( 29 ). Studies have identified genetic variants that participate in complex interactions between genes and nutritional factors responsible for influencing weight and body composition. In this context, nutrigenetics, which is the study of the effect of genetic variation on an individual’s nutritional needs, can potentially improve the understanding of weight control and contribute to personalized dietary management of obesity ( 30 ).

Obesity is a multifactorial and polygenic condition and represents a significant public health issue in both developed and developing countries. Cardiovascular disease, T2D, non-alcoholic fatty liver disease, metabolic syndrome, and cancer are among the leading health issues accounting for morbidity associated with an increased prevalence of obesity. From the 1990s onwards, it has been possible evidence of how obesity influences inflammatory conditions, which are directly involved in the etiology of cardiovascular disease, T2D, and certain types of cancer ( 31 ).

Obesity can be either monogenic, meaning it can be caused by genetic variations in either a single gene or in a specific disease-related chromosomal region, or polygenic, where the sum of SNPs in several genes (each accounting for a minimal effect) determine an individual’s weight. Many genes associated with obesity are involved in regulating energy intake, lipid metabolism, adipogenesis, thermogenesis, adipocytokine synthesis, and transcription factors ( 32 ).

Importantly, the genetic basis of polygenic obesity is diffuse, multifactorial, and non-deterministic. Many variants are spread throughout the genome and have a small contribution to obesity onset – thus making it a challenge for clinical practice. A set of genetic variant information is needed to characterize susceptibility to obesity ( 33 ). For this reason, several authors have employed the “polygenic risk scores” or “genetic risk scores” (GRS) based on the sum of the number of risk alleles, sometimes multiplied by their effect sizes ( 34 - 36 ). These polygenic scores have been useful for risk assessment in various diseases ( 37 , 38 ), including metabolic syndrome ( 39 ), and obesity ( 47 - 49 ). Estimating an individual’s susceptibility to a disease can be a powerful tool for prevention and treatment if well-communicated and understood ( 40 ).

Regarding adiposity, advances in nutrigenetics have sought to determine the interaction of nutritional and genetic factors that affect body fat deposition ( 41 - 43 ). The design of nutrigenetic studies can involve an analysis of complete dietary patterns ( 44 ), in which the MedDiet stands out amongst the most studied ones.

A study with a sample of Iranian individuals, using GRS from six *FTO* polymorphisms showed that higher adherence to the MedDiet decreased the risk of developing obesity in individuals with higher risk alleles when compared to those with a lower diet adherence and a lower genetic susceptibility to obesity. These results highlight the beneficial effects of this dietary pattern ( 45 ). These results are in accordance with the ones from other reports ( Table 2 ).

**Table 2:** Summary of studies investigating the effect of interactions between the Mediterranean diet and genetic variants on obesity and obesity-related traits

Reference	Genes	Type of genetic variant	Study design	Population (n)	Phenotype	Diet	Interaction
Razquin et al. ( 134 )	ADIPOQ	SNP (rs1501299 G>T)	RCT	Spain, high cardiovascular risk, adults (737)	Delta-Body weight	3-years intervention with the Mediterranean diet vs. Control diet	Men with the TT genotype had higher body weight gain (vs. G allele carrier) after the intervention. The Mediterranean diet canceled the effect of genotype on body weight gain.
Razquin et al. ( 134 )	ADIPOQ	SNP (rs2241766 T>G)	RCT	Spain, high cardiovascular risk, adults (737)	Delta-Body weight	3-years intervention with the Mediterranean diet vs. Control diet	No interactions were found
Sánchez-Moreno et al. ( 135 )	APOA5	SNP (rs662799 T>C)	Cross-sectional	Spain, overweight or obese, adults (1465)	BMI (kg/m ^2^ ), body fat (%), Waist circumference (cm), hip (cm)	Macronutrient profile of the diet	Only TT carriers had a higher BMI, waist and hip circumferences when their diets were high in total fat and saturated fat.
Garaulet et al. ( 136 )	CLOCK	SNP (rs4580704 C>G)	Non-RCT	Spain, overweight or obese, adults (454)	Weight (kg), BMI (kg/m ^2^ ), body fat (%), waist and hip circumference (cm)	28-weeks hypocaloric Mediterranean diet advice and maintenance protocol	No interactions were found
Garaulet et al. ( 136 )	CLOCK	SNP(rs1801260 A>G)	Non-RCT	Spain, overweight or obese, adults (454)	Weight (kg), BMI (kg/m ^2^ ), body fat (%), waist and hip circumference (cm)	28-weeks hypocaloric Mediterranean diet advice and maintenance protocol	G allele carriers had higher BMI at baseline and were less successful in losing weight after the intervention period.
Garaulet et al. ( 136 )	CLOCK	SNP(rs3749474 C>T)	Non-RCT	Spain, overweight or obese, adults (454)	Weight (kg), BMI (kg/m ^2^ ), body fat (%), waist and hip circumference (cm)	28-weeks hypocaloric Mediterranean diet advice and maintenance protocol	No interactions were found.
Corella & Ordovas ( 137 )	FAIM2	SNP (rs7138803 G>A)	RCT	Spain, high cardiovascular risk, adults (7161)	BMI (kg/m ^2^ ), body weight (kg), and waist circumference (cm)	4.8-years intervention with the Mediterranean diet vs. Control diet	No interactions were found
Hosseini-Esfahani, et al. ( 45 )	FTO	SNP(rs1121980 G>A)	Nested case-control study	Iran, adults (627 cases and 1,254 controls)	Obesity (BMI cut-off) and abdominal obesity (waist circumference and waist-to-hip ratio cut-offs)	Mediterranean diet score	No interactions were found
Hosseini-Esfahani, et al. ( 45 )	FTO	SNP(rs1421085 T>C)	Nested case-control study	Iran, adults (627 cases and 1,254 controls)	Obesity (BMI cut-off) and abdominal obesity (waist circumference and waist-to-hip ratio cut-offs)	Mediterranean diet score	No interactions were found
Hosseini-Esfahani, et al. ( 45 )	FTO	SNP(rs1781749 T>G)	Nested case-control study	Iran, adults (627 cases and 1,254 controls)	Obesity (BMI cut-off) and abdominal obesity (waist circumference and waist-to-hip ratio cut-offs)	Mediterranean diet score	Only G allele carriers had decreased odds for obesity and abdominal obesity when in the highest quartiles of the Mediterranean diet score.
Hosseini-Esfahani, et al. ( 45 )	FTO	SNP(rs3751812 G>T)	Nested case-control study	Iran, adults (627 cases and 1,254 controls)	Obesity (BMI cut-off) and abdominal obesity (waist circumference and wais-to-hip ratio cut-offs)	Mediterranean diet score	Only T allele carriers had decreased odds for obesity and abdominal obesity when in the highest quartiles of the Mediterranean diet score.
Hosseini-Esfahani, et al. ( 45 )	FTO	SNP(rs8050136 G>A)	Nested case-control study	Iran, adults (627 cases and 1,254 controls)	Obesity (BMI cut-off) and abdominal obesity (waist circumference and wais-to-hip ratio cut-offs)	Mediterranean diet score	Only A allele carriers had decreased odds for obesity and abdominal obesity when in the highest quartiles of the Mediterranean diet score.
Corella et al. ( 138 )	FTO	SNP(rs9939609 C>A)	Cross-sectional	Spain, high cardiovascular risk, adults (7,052)	BMI (kg/m ^2^ ), body weight (kg), and waist circumference (cm)	Adherence do the Mediterranean diet	No interactions were found
Roswall et al. ( 139 )	FTO	SNP(rs9939609 C>A)	Nested case-control study	Europe, adults (5,552 cases and 5,496 controls)	Delta-Body weight, and Delta-Waist circumference	Mediterranean diet score	No interactions were found
Razquin et al. ( 134 )	FTO	SNP(rs9939973 G>A)	RCT	Spain, high cardiovascular risk, adults (776)	BMI (kg/m ^2^ ), body weight (kg), and waist circumference (cm)	3-years intervention with the Mediterranean diet vs. Control diet	A allele carriers had the lowest body omozy gain (vs. GG genotype) after the intervention with the Mediterranean diet. This difference was not observed in individuals omozygou to the control diet intervention.
Hosseini-Esfahani, et al. ( 45 )	FTO	SNP(rs9939973 G>A)	Nested case-control study	Iran, adults (627 cases and 1,254 controls)	Obesity (BMI cut-off) and abdominal obesity (waist circumference and wais-to-hip ratio cut-offs)	Mediterranean diet score	Only A allele carriers had decreased odds for obesity when in the highest quartiles of the Mediterranean diet score.
Corella et al. ( 138 )	MC4R	SNP(rs17782313 T>C)	Cross-sectional	Spain, high cardiovascular risk, adults (7,052)	BMI (kg/m ^2^ ), body weight (kg), and waist circumference (cm)	Adherence do the Mediterranean diet	No interactions were found
de Luis et al. ( 140 )	MTNR1B	SNP(rs10830963 C>G)	Non-RCT	Spain, obese, adults ( 80 )	BMI (kg/m ^2^ ), body weight (kg), fat mass (g), waist circumference (cm)	12-weeks intervention wih hypocaloric Mediterranean diet	The improvement of the anthropometric parameters after the intervention was higher among CC cariers (vs. G allele carriers).
Garaulet et al. ( 141 )	PPARG	SNP(rs1801282 C>G)	Non-RCT	Spain, overweight or obese, adults	Delta-Body weight	Behavioural treatment program for obesity based on a Mediterranean diet (duration varied according to patients need)	G allele carriers had less weight loss when fat intake was above 42.6% of total energy intake, in comparison do CC homozygotes.
Roswall et al. ( 139 )	TCF7L2	SNP(rs7903146 C>T)	Nested case-control study	Europe, adults (5,552 cases and 5,496 controls)	Delta-Body weight, and Delta-Waist circumference	Mediterranean diet score	Only when the Mediterranean diet score was high, there was a lower weight gain in TT omozygous, in comparison to C allele carriers.
Barchitta et al. ( 142 )	TNF	SNP(rs1800629 G>A)	Cross-sectional	Italy, women only, adults (380)	Overweight and obesity (BMI cut-off)	Mediterranean diet score	No interactions were found
Corella et al. ( 138 )	FTO, MC4R	2-SNP GRS	Cross-sectional	Spain, high cardiovascular risk, adults (7,052)	BMI (kg/m ^2^ ), body weight (kg), and waist circumference (cm)	Adherence do the Mediterranean diet	No interactions were found
Garaulet et al. ( 143 )	CLOCK, SIRT1	2-SNP GRS	Non-RCT	Spain, overweight or obese, adults (1,465)	Delta-Body weight (kg)	40-weeks intervention with dietary advice to loose weight, based in the Mediterranean diet	Individuals with higher GRS lost less body weight than individuals with lower GRS after the intervention period
Frankwich et al. ( 144 )	APOA2, ADIPOQ, FTO, KCTD10, LIPC, MMAB, PPARG	7-SNP GRS	RCT	US, men only, adults ( 51 )	proportion of individuals achieving at least 5% body weight loss	8-weeks and 24-weeks genetic-guided dietary advice vs. Standard dietary advice	No difference was observed between the interventions
Hosseini-Esfahani et al. ( 45 )	FTO	6-SNP GRS	Nested case-control study	Iran, adults (627 cases and 1,254 controls)	Obesity (BMI cut-off) and abdominal obesity (waist circumference and wais-to-hip ratio cut-offs)	Mediterranean diet score	The odds for obesity decreased across quartiles of the Mediterranean diet score only in individuals with GRS ≥ 6. No interaction was found for abdominal obesity traits.
San-Cristobal et al. ( 36 )	ADRB2, APOA5, APOE, BCMO1, COMT, GC, GPX1, MTHFR, SLCA4, SOD2, TCF7L2, TPH2, VDR	14-SNP GRS	RCT	Europe, adults (1,263)	BMI (kg/m ^2^ ) and waist circumference (cm)	6-month personalized dietary advice based in Mediterranean diet	No interactions were found.
Ding et al. ( 145 )	Near 158 genes associated with BMI	97-SNP GRS	3-Longitudinal cohorts (pooled)	US, adults (31,058)	BMI (kg/m ^2^ )	Alternative Mediterranean diet score	Individuals in the highers tertile of the alternative Mediterranean diet score had lower increases in BMI per 10 risk allele increase of the GRS
Ding et al. ( 145 )	Near 102 genes associated with BMI and highly expressed in central nervous system	54-SNP GRS	3-Longitudinal cohorts (pooled)	US, adults (31,058)	BMI (kg/m ^2^ )	Alternative Mediterranean diet score	Individuals in the highers tertile of the alternative Mediterranean diet score had lower increases in BMI per 10 risk allele increase of the GRS
Ding et al. ( 145 )	Near 56 genes associated with BMI and highly expressed in tissues other than the central nervous system	43-SNP GRS	3-Longitudinal cohorts (pooled)	US, adults (31,058)	BMI (kg/m ^2^ )	Alternative Mediterranean diet score	No interactions were found
Hennein et al. ( 146 )	Intergenic, long non-coding RNA, regulatory regions	4-SNP GRS	Longitudinal cohort	US, adults (1,677)	pericardial fat depots	Mediterranean diet score	Only individuals in the high-GRS cathegory had increased pericardial fat depot when Mediterranean diet score decreased during the follow-up period
Hennein et al. ( 146 )	Intergenic, long non-coding RNA	2-SNP GRS	Longitudinal cohort	US, adults (1,677)	visceral fat depots	Mediterranean diet score	No interactions were found.
Hennein et al. ( 146 )	FTO, ATXN1	3-SNP GRS	Longitudinal cohort	US, adults (1,677)	subcutaneous fat depots	Mediterranean diet score	No interactions were found.

BMI: body mass index; GRS: genetic risk score; RCT: randomized clinical trial; SNP: single nucleotide polymorphism. The same study was included more than once when more than one SNP was evaluated separately.

In this context, genetic factors are not only responsible for 45%-75% of interindividual variations in Body Mass Index (BMI) ( 46 ), and adiposity (which can be influenced at a rate of 75%-80%) ( 47 ), but weight loss in response to dietary interventions is also determined by genetic variants ( 35 , 48 ). The different strategies used in the treatment of obesity can result in significant weight loss; however, the individual response is variable, and it is possible to identify the hypo- or hyper-responders to specific treatments ( 49 , 50 ). Thus, according to the literature, genetic variations, including SNPs, may at least in part explain this interindividual variation in response to a dietary pattern, including the MedDiet ( 44 ).

As mentioned before, the GRS calculation includes a combination of different genetic variants at the same time. Although studies of interactions between genes and MedDiet are initial, some results are promising and suggest that individuals with greater genetic susceptibility to certain diseases may benefit from the effects of this diet ( 51 , 52 ). Corella & Ordovas ( 53 ) were pioneers in establishing how diet can modulate the genetic risk of the disease, and several authors have been studying the association between MedDiet and genetic variants in different populations and phenotypes, including obesity ( Table 2 ).

Ortega-Azorin and cols. ( 54 ) investigated the influence of MedDiet and the effects of T2D risk alleles on rs9939609 SNPs for the *FTO* gene and rs17782313 for the *MC4R* gene. The authors demonstrated that individuals with both poor adherence to the diet and allelic variants of risk had a higher risk of disease development. On the other hand, the same variants for both genes – alone or in combination – were no longer related to a higher risk of disease development when dietary compliance was high.

A study with Puerto Rican individuals living in Boston, USA, with risk genotypes for T2D located in the transcription factor 7-like 2 (TCF7L2) gene (rs7903146 and rs12255372), showed a better anthropometric profile under complete adherence to the MedDiet. This suggested that unfavorable genetic predisposition can be offset by a healthy diet. Moreover, haplotype analysis based on the combination of two risk alleles showed that individuals with higher genetic risk had lower BMI when adhering to the MedDiet ( 55 ).

San-Cristobal and cols. ( 36 ) evaluated associations and possible interactions between adherence to the MedDiet and the genetic background of the Food4Me study. The authors developed a GRS from risk alleles and a MedDiet score (DMS) based on food intake data. At the baseline, there were no correlations between scores and metabolic characteristics. However, after 6 months, there was a significantly greater decrease in total cholesterol in individuals with low GRS when compared to those with high GRS. In addition, a high DMS was linked to greater reductions in BMI, waist circumference, and blood glucose. This suggested that increased dietary compliance induces beneficial effects on metabolic outcomes, which may be affected by the genetic profile in some specific markers.

Within this context, studies on the interactions between genes and diet can better elucidate the heterogeneity of responses to dietary interventions, showing that these responses are often individual. Importantly, the MedDiet has interactions with several genes that act in both obesity-related pathways and other associated diseases, including cardiovascular disease, T2D, and cancer ( 51 ).

The authors highlighted that some existing results are promising and suggested that individuals with greater genetic susceptibility to certain diseases may benefit from the effects of the MedDiet, thus making it extremely useful for precision nutrition ( 52 ). A recent review has also presented an up-to-date view of the influence of the MedDiet on different phenotypes with associated diseases, including cardiovascular, neurodegenerative, cancer, and obesity. The report shows that studies involving Mediterranean gene-diet interactions are extraordinarily complex and scarce ( 51 ). In Brazil, there are also few studies evaluating the association between polymorphisms and MedDiet ( 56 , 57 ).

Therefore, one of the biggest challenges of nutritional genomics in obesity, besides integrating all information from the “omic” strands (genomics, metabolomics, proteomics, and transcriptomics), is to extrapolate the findings on the interactions between genes and diet for different populations ( 58 - 60 ). There is already evidence that suggests the reproducibility of these genomic results in various cohorts ( 60 , 61 ). Large-scale studies with replication in varying populations are likely required to provide significant and detailed evidence, including the various types of weight-loss interventions, phenotypes, obesity genetic risk scores, and genetic variants that determine eating preferences and behaviors.

In the context of gene-diet interaction and inflammation, variants located in *ADIPOQ, CRP, TNF* , and *APOE* genes are the most frequently studied, and the ones for which interaction with diet has been tested in more than one population.

The *ADIPOQ* gene encodes for the adiponectin protein, secreted mainly in adipose tissue. This protein has critical hormonal functions in the muscle, liver, adipose tissue, hypothalamus, and vasculature cells, where it exerts anti-inflammatory, antioxidant, and insulin sensitizer effects ( 62 ). Accordingly, low adiponectin blood levels have been related to metabolic inflammation-related diseases, such as metabolic syndrome, T2D, and cardiovascular diseases ( 63 ).

Adiponectin blood levels are a heritable trait (heritability ranging from 42%-88%) ( 64 - 67 ). Specifically, *ADIPOQ* SNP, rs1501299 G>T, located in intron 2, which is in linkage disequilibrium with rs2241766 T>G, located in exon 2, has been positively and inversely linked to adiponectin blood levels depending on the studied population ( 68 , 69 ). Concerning these two *ADIPOQ* genetic variants, the number of studies aiming to investigate the influence of their interaction with the MedDiet or its components on inflammation has increased. For instance, a cross-sectional study conducted with a representative sample of Greek children found an association between dietary fiber intake and rs1501299 G>T influencing serum adiponectin concentration. It should be noted that the T allele carriers had lower adiponectin concentration. However, when dietary fiber was above the highest tertile of intake, the effect of the *T* allele was no longer observed ( 70 ). Furthermore, a randomized clinical trial with Spanish obese adults observed an increase in serum adiponectin concentration after a 9-month intervention with hypocaloric diets only in *GG* homozygotes for the rs1501299 SNP ( 71 ). However, no similar interactions were observed in other populations ( 72 - 74 ).

*ADIPOQ* rs2241766 T>G, in turn, seems to interact with omega-3 fatty acids not only impacts serum adiponectin concentration but other inflammatory biomarkers as well ( 72 , 74 ). In a cross-sectional population-based study, an adult Brazilian population was dichotomized into two clusters according to the plasma concentration of eleven inflammatory biomarkers. The authors found that a higher total plasma omega-3 fatty acids content was protective against inflammation only in G allele carriers of the rs2241766 SNP ( 72 ). Likewise, in a randomized, controlled trial with UK adults, TT homozygotes for the rs2241766 had a decrease in serum adiponectin concentration after a 12-month intervention with daily intakes of 0.9 g of highly unsaturated omega-3 fatty acids (EPA and DHA)

CRP is an acute-phase protein that has been extensively used as an important inflammatory marker, in addition to being a cardiovascular risk indicator ( 75 ). The *CRP* rs1205 T>C SNP, located in the untranslated region 3’, has been associated with higher levels of blood CRP in many populations ( 76 - 80 ). In a clinical trial with 1,584 US adults, the higher plasma CRP concentration of *CC* homozygotes *vs. T* allele carriers at baseline was no longer observed after 12 months following personalized healthy lifestyle advice (dietary advice based in the Dietary Approach to Stop Hypertension) ( 77 ). Similarly, in a cross-sectional population-based study with Brazilian adults, only the *T* allele carriers had lower odds for low-grade systemic inflammation when their highly unsaturated omega-3 fatty acid plasma content was above the median ( 80 ). Nevertheless, some studies have not found any associations between rs1205 and diet components ( 78 , 79 ), nor MedDiet ( 76 ), in other populations.

For the *TNF* gene, no other SNP stands out as much as rs1800629 G>A. Located in the *TNF* gene promoter region, the *AA* genotype for the SNP has been associated with higher levels of its encoding protein – the pro-inflammatory cytokine TNF-α – in addition to auto-immune and inflammatory diseases susceptibility ( 81 ). Furthermore, when the gene-diet interaction was investigated for this SNP, only *GG* homozygotes had a decrease in plasma CRP concentration after a 12-month intervention with MedDiet in metabolic syndrome patients from Spain ( 82 ). In a study with Canadian adults, the same decrease in plasma CRP concentration was observed in *GG* carriers after a 6-week supplementation with fish oil ( 79 ). Moreover, in a Brazilian study group, only A allele carriers had higher odds for an inflammatory cluster when both plasma stearic acid and total saturated fatty acid contents were higher overall ( 83 ).

In contrast to other genes cited so far, the *APOE* gene does not encode for a protein directly involved with inflammation, but rather encodes for the apolipoprotein E. This protein is mainly related to lipids and lipoprotein metabolism ( 84 ). Recently, novel functions for the apolipoprotein E have emerged: anti-inflammatory properties, anti-platelet aggregation, and maintenance of the mitochondrial function ( 84 ). Two SNP located in the coding region of *APOE* gene (rs7412 and rs429358) modify apolipoprotein E mRNA codon 112 and 158, respectively. In this case, cysteine is replaced by arginine in both positions, and hence forms three possible isoforms of the protein according to the resultant salt-bridges: ε *2* , ε *3* , and ε *4* . Therefore, the six possible genotype combinations are ε *2/* ε *2,* ε *2/* ε *3* , ε *2/* ε *4,* ε *3/* ε *3,* ε *3/* ε *4* , and ε *4/* ε *4* . However, the combinations ε *2/* ε *2* , ε *2/* ε *4* , and ε *4/* ε *4* are very rare ( 84 ). *APOE* ε *4* carriers have a higher risk for Alzheimer’s disease, while the APOE ε *2* genotype has been associated with longevity ( 84 ).

Studies have shown that *APOE* ε *2* and ε *3/* ε *3* genotypes have been associated with higher plasma CRP concentration and that these variants interact with dietary components in the context of inflammation ( 85 - 87 ). In a cross-sectional study with 4,265 US adults, only *APOE* ε4 carriers had higher plasma CRP concentrations with higher intakes of alcoholic beverages ( 85 ). Likewise, in a crossover clinical trial conducted in 176 British adults, only *APOE* ε *4* carriers showed an increase in plasma CRP after eight weeks of a high-saturated fatty acid diet ( 86 ). In another UK sample, there was a reduction in plasma CRP concentration only in *APOE* ε *4* carriers, as opposed to an observed increase in *APOE* ε *3/* ε *3* carriers after a 16-week dietary intervention with the substitution of 9.5% energy from saturated fatty acid with monounsaturated or omega-6 fatty acids ( 87 ). Thus, for *APOE* ε *4* carriers, the recommendation of reducing saturated fatty acid intake (possibly substituting it with other unsaturated fatty acids) appears to be protective against metabolic inflammation in the British population.

Recently, a CRP-GRS was developed to sum up the effect of 20 SNP with the strongest association with plasma CRP concentration ( 88 ). The CRP-GRS explains 4%-5% of the variation in plasma CRP – more than twice the effect of the strongest associated SNP alone ( *APOC1* rs4420638) ( 88 ). However, no GRS for metabolic inflammation has been tested so far in relation to its interaction with dietary components. Therefore, studies aiming to test the relationship between diet and GRS for inflammation are needed and may be a subject for future investigations.

## NUTRITIONAL EPIGENOMICS, INFLAMMATION, AND OBESITY

Epigenetics are reversible alterations in gene expression that do not involve changes in DNA sequence. The most studied epigenetic modifications are DNA methylation, covalent histone modifications, and microRNAs (miRNAs). Epigenetic mechanisms can silence genes, regulate gene expression, and modify chromatin architecture ( 89 ).

Epigenetic modifications are related to metabolic diseases such as obesity, metabolic syndrome, and T2D. These modifications modulate critical genes involved in appetite regulation, adipogenesis, glucose homeostasis, body weight, inflammatory response, and lipid storage. For example, the promoter of the *PPARG* gene – a key transcriptional regulator of adipogenesis – is hypermethylated in 3T3-L1 preadipocytes but is demethylated upon induction of differentiation. Furthermore, the expression of the insulin gene is regulated by cytosine methylation, which can contribute to the development of T2D ( 90 ). Nutrients can regulate DNA methylation and histone modifications by directly inhibiting epigenetic enzymes or changing the availability of substrates required for the enzymatic reactions ( 89 ). The MedDiet effects on epigenetic modifications were investigated by Arpón and cols. ( 91 ), who followed subjects from the PREDIMED study for five years. The MedDiet was linked to the differential methylation of inflammation-related genes such as *EEF2, COL18A1, IL4I1, LEPR, PPARGC1B, MAPKAPK2, IFRD1* , and *PLAGL1* in peripheral blood cells. The authors concluded that the MedDiet could exert an anti-inflammatory activity that might be mediated by epigenetic mechanisms. A Greek study evaluated the effects of the ratio of PUFA to SFA, the ratio of MUFA to SFA, and the ratio of PUFA+MUFA to SFA on genome-wide DNA methylation pattern in whole peripheral blood of eutrophic and obese children. DNA methylation was related more to the quality than to the quantity of fat intake. In this, omega-3 (n-3) PUFA showed a contribution to histone modifications involved in leptin regulation – a pro-inflammatory adipokine – on obesity ( 92 ).

miRNAs are involved in several diseases, and their imbalance may play a role in the development of obesity and other related metabolic complications. In this review concerning nutritional epigenomics, we have focused on the functions of miRNAs in their relationship with obesity and inflammation ( 93 , 94 ).

### miRNAs: biogenesis and biological function

miRNAs are non-coding endogenous RNA molecules (~18-25 nucleotides) that are involved in post-transcriptional gene regulation by binding to the 3’ untranslated region (UTR) of a target messenger RNA (mRNA), resulting in degradation or inhibition of translation ( 95 ). miRNAs can also bind to the 5’-UTR, or coding region, and activate, rather than suppress, mRNA translation ( 96 ).

Other roles of miRNAs are also described in the literature. miRNAs can modulate the transcriptional processes by interfering in histone and DNA methylation, where they target vital enzymes responsible for epigenetic reactions. These key enzymes are the following: DNA methyltransferases (DNMTs); methylation-related proteins, including methyl CpG binding protein 2 (MeCP2) and methyl-CpG binding domain proteins 2 and 4 (MBD2 and MBD4); histone deacetylases (HDACs); and histone methyltransferases (EZH) ( 97 , 98 ). Furthermore, miRNAs can downregulate other types of RNAs that are responsible for inhibiting transcription to then increase gene expression ( 99 , 100 ).

**Figure 1:**
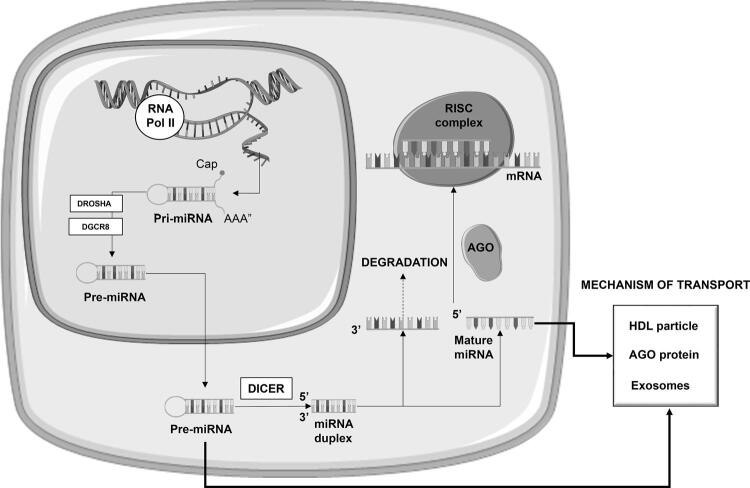
MicroRNA biogenesis and cellular release mechanisms. MicroRNAs (miRNA) is transcribed by RNA polymerase II from miRNA genes, first forming the ‘primary miRNA transcript’ (pri-miRNA), which is then cleaved by the DROSHA/ DiGeorge syndrome critical region 8 (DGCR8) microprocessor complex to form the ‘miRNA precursor’ (pre-miRNA). Pre-miRNA is then exported from the nucleus to the cytoplasm by exportin 5 and further processed by DICER to originate the mature miRNA. Mature miRNA is loaded into the miRNA-induced silencing complex (miRISC), which contains Argonaute (AGO) proteins, that targets mRNA by sequence complementary binding and mediates gene suppression by targeted mRNA degradation. The cellular release mechanisms include pre-miRNA or mature miRNA associated to RNA-binding proteins, such as Ago2 or their binding to high-density lipoproteins (HDL). Furthermore, pre-miRNA or mature miRNA can be incorporated into small vesicles called exosomes, which are extracellular vesicles of endosomal origin that have emerged as key mediators of intercellular communication.

The biogenesis of miRNAs occurs through a sequential process that involves a variety of enzymes and proteins ( 101 ). miRNAs biogenesis is shown in Figure 1 . Under most conditions, the mature RNA-induced silencing complex (RISC) represses gene expression post-transcriptionally. This occurs by binding the 3’-UTR of specific mRNAs and mediating mRNA degradation, destabilization, or translational inhibition according to sequence complementarity to the target ( 101 - 104 ).

miRNAs act not only within cells but also as hormones controlling gene expression in distant tissues. This is achieved through the transport of secreted miRNA by membrane-vesicles as exosomes (extracellular vesicles of endosomal origin), or bound to lipoproteins (LDL or HDL), proteins, apoptotic bodies, and ribonucleoprotein complexes (linked to Argonaut) ( 105 , 106 ). miRNAs are present in both tissues and body fluids, such as plasma, serum, urine, and saliva, in which they are in a stable form and protected from endogenous RNAse activity ( 107 ). About 10% of all human miRNAs’ particles can be found in plasma, and are called circulating miRNAs ( 108 ).

The importance of regulating gene expression through miRNA is highlighted because a single miRNA can act on several target genes, and the same mRNA can pair with different miRNAs. Thus, according to Friedman and cols. ( 109 ), more than 60% of the human genes can be regulated by miRNA.

### microRNAs and inflammation in obesity

Changes in miRNAs levels have been shown in several pathophysiological disorders related to obesity. These include disorders such as inflammation, oxidative stress, impaired adipogenesis, insulin signaling, apoptosis, and angiogenesis ( 110 - 114 ). miRNAs can act as potential diagnostic biomarkers since they are rapidly and accurately detected by non-invasive methods. As such, they allow for early detection and have a long half-life in the sample ( 115 - 117 ).

In this context, Lorente-Cebrián and cols. ( 118 ) verified that obese individuals have a different miRNA expression plasma profile when compared to eutrophic individuals. This indicates the possible role of miRNAs in metabolic inflammation. Such inflammation is characterized by a chronic, systemic, low-intensity inflammation which differs from that caused by external agents ( *e.g.* , bacterial infection and tissue injury) ( 24 ). For example, obese individuals have lower miR-145 plasma levels than lean individuals, and, regarding the inflammatory process mediated by this miRNA, a higher expression of both TNF-α and IL-6 in white adipose tissue (WAT) was observed when this miRNA was downregulated ( 119 , 120 ). miR-181a-5p and miR-23a-3p were reduced in adipose tissue from obese subjects, and its overexpression contributed to TNF-α downregulation in visceral WAT ( 121 ). miRNAs can modulate the inflammation in adipose tissue by regulating macrophage activation. Treatment with miR-10a-5p was associated with altering macrophage polarization to an anti-inflammatory phenotype ( 121 , 122 ).

Overweight subjects without T2D at baseline from CORDIOPREV trial were followed by four years to evaluate whether plasma miRNAs were related to the risk of T2D. This study showed that deregulated plasma levels of miR-150, miR-30a-5p, miR-15a, and miR-375 were observed years before the onset of T2D and pre-diabetes. In this way, these miRNAs could be used to evaluate the risk of developing the disease, which may improve prediction and prevention among individuals at high risk for T2D ( 123 ).

### Diet and microRNAs modulation

Nutrimiromics describes the influence of diet on the modification of gene expression. The term refers specifically to the epigenetic processes relating to miRNAs that influence an individual’s risk of developing chronic diseases ( 104 ).

Nutrients and bioactive compounds of food can modulate the miRNAs expression, regulating inflammation in the WAT of obese subjects. Within bioactive compounds, polyphenols receive special attention, although the mechanism involved in this regulation is not precise. One hypothesis is that polyphenols could influence miRNA functionality by changing its binding to mRNA related to the target gene. Polyphenols could also bind to a component of miRNA biogenesis ( 7 ).

An interventional study with healthy Brazilian women showed that miR-145a-5p – which is related to the inflammatory pathway – was altered in the postprandial period after a single intake of a high-fat meal rich in SFA. This is an example of a potential biomarker for a Western diet pattern and its effect on inflammation ( 124 ).

In a randomized, placebo-controlled study, the authors observed modulation of miRNAs after treating 35 diabetic hypertensive men. The treatment was undertaken for a one-year period and involved administering a grape extract containing 8 mg of resveratrol. According to the author, these miRNAs are related to a regulatory role in inflammatory responses. Upregulation was observed for miR-21, miR-181b, miR-663, and miR-30c2, whereas others such as miR-34a and miR-155 were downregulated in PBMC ( 125 ).

Ortega and cols. ( 126 ) showed that an intake of nuts (30 g/day of almonds and walnuts) modulated the expression of plasma miRNAs. Nuts downregulated the expression of miR-328, miR-330-3p, miR-221, and miR-125a-5p, and upregulated the expression of miR-192, miR-486-5p, miR-19b, miR-106a, miR-769-5p, miR-130b, and miR-18a in obese subjects. Also, miR-130b and miR-221 were related to a reduction in plasma CRP levels. The downregulation of miR-125a-5p was linked to a reduction of plasma triacylglycerols and increased adiponectin levels. The effect of nuts on circulating miRNA expression was also shown in a very recent study in which obese women consumed Brazil nut for two months. The intake of Brazil nut upregulated the expression of miR-454-3p and miR-584-5p ( 127 ).

Recently, a clinical trial ( 128 ) showed the effect of a hypoenergetic diet based on MedDiet (30% energy restriction) for eight weeks in subjects with metabolic syndrome. Nutritional intervention downregulated the expression of miR-155-3p in white blood cells and upregulated the let-7b expression. The increased expression of let-7b was linked to a low intake of lipids and saturated fats.

Current studies that link nutrition to miRNAs in humans are scarce and do not show a cause-effect relationship. Thus, further studies are needed to elucidate the molecular mechanism by which nutrients and bioactive compounds modulate miRNA expression and the metabolic pathways affected by miRNAs ( 129 ).

We conclude that the search for nutritional biomarkers for applications in clinical practice remains a challenge. However, these findings will allow for the early diagnosis of diseases, facilitate appropriate interventions, and even predict responses to different types of treatment ( 83 ). In recent years, great efforts have been made to identify the biomarkers that may influence the treatment of the obesity-related inflammatory process. The knowledge gained from nutritional genomics requires an evidence-based approach for personalized recommendations to be validated and proven beneficial for individuals ( 7 , 84 ).

Despite the great deal of progress made so far, this is a relatively new field, and the use of nutrigenetic tests requires careful attention from professionals with deep knowledge, ethics, and experience. In addition, studies aiming at investigating gene-diet interaction in the context of inflammation are mainly candidate-gene studies, and all polymorphisms that have been investigated in at least two independent samples still need replication. This lack of replication is often attributed to the large variation in study designs and the small effect of a single genetic variant on complex outcomes, such as inflammation. Therefore, it is important to highlight the use of GRS as an important tool in the application, handling, and administration of personalized nutrition (identifying a diet based on this score), thus bringing significant benefits to the obese.
